# Mold Surface Optimization and Process Parameter Investigation for Preforming in Advanced Pultrusion of Composite Structures

**DOI:** 10.3390/polym18101244

**Published:** 2026-05-20

**Authors:** Mengting Sun, Zongsu Zhang, Feng Liu, Qigang Han

**Affiliations:** 1 Key Laboratory of Automobile Materials, Ministry of Education, School of Materials Science and Engineering, Jilin University, Changchun 130022, China; sunmt23@mails.jlu.edu.cn; 2 State Key Laboratory of Advanced Forming Technology and Equipment, China Academy of Machinery Science and Technology, China Academy of Machinery Science and Technology Group Co., Ltd., Beijing 100044, China; zhangzongsu99@126.com; 3 Institute of Structured and Architected Materials, Liaoning Academy of Materials, Shenyang 110167, China; 4 Weihai Institute for Bionics, Jilin University, Weihai 264207, China

**Keywords:** advanced pultrusion, preforming process, mold surface optimization, process parameters, composite materials

## Abstract

Advanced pultrusion technology for composite materials is an automated forming process that uses pre-impregnated materials as raw materials and is oriented towards the manufacturing of continuous components. It is particularly suitable for the continuous manufacturing of ultra-long components with uniform cross-sections and has a promising application prospect in the field of aviation composite materials. However, during the preforming stage, the pre-impregnated materials are prone to strain concentration and uneven thickness under the constraint of the mold surface, and in severe cases, there is a tendency to form wrinkles. Moreover, the severity of these defects is further influenced by the process parameters. In response to the above problems, this paper proposes a mold surface optimization method based on the finite element model with the goal of three-dimensional strain homogenization, which controls the thickness direction and in-plane strain within 5%, effectively improving the material deformation coordination. Furthermore, the influence law of preforming temperature, traction speed and tension on preforming quality was systematically analyzed through experimental research. It was found that the influence of each process parameter on appearance quality, thickness uniformity and internal quality all showed a trend of “improvement first and then deterioration”, thus obtaining a relatively better combination of process parameters for preforming quality. The results of this study provide methodological and technical support for the research on advanced pultrusion preforming processes of complex cross-section components.

## 1. Introduction

Composite materials, as highly engineered materials, are widely used in fields such as aerospace [1,2,3]. Due to its ability to significantly reduce weight and directly save fuel, composite materials for aircraft have gradually evolved from being initially used in non-load-bearing components to being applied in secondary and primary load-bearing components, and the proportion is gradually increasing [4]. In this context, achieving high-quality, low-cost, and large-scale manufacturing of composite material components has become an important research direction at present.

The composite material beam structure serves as the core load-bearing component of the fuselage and wing stiffening panels. Its manufacturing directly affects the service performance and production cost of the aircraft. Currently, such components are mainly produced using the autoclave molding process, which has problems such as high investment, high energy consumption, low efficiency, and limited part size [5,6]. It is difficult to adapt to the development trend of continuous and large-scale manufacturing of composite material structures. In contrast, advanced pultrusion (ADP) using prepreg as the raw material [7,8,9,10] is considered a potential process for achieving low-cost and large-scale production of beam structure components. Compared with traditional pultrusion molding, ADP uses prepreg as the raw material, which makes the resin content and fiber distribution more controllable, the layup design more flexible, and effectively improves the insufficient transverse mechanical properties of traditional pultrusion [11,12,13,14] products.

During the ADP forming process, the preforming stage plays a crucial role in transforming the flat pre-impregnated laminate into the desired cross-section. The deformation outcome of this stage directly determines the initial geometric shape and internal state of the material before it enters the subsequent hot pressing curing stage. In this stage, the pre-impregnated material undergoes complex fiber rearrangement and geometric deformation under the constraints of the mold surface and the pulling force. If defects such as fiber buckling, wrinkling, and uneven thickness occur during this stage, they tend to be “inherited” in subsequent processes and are generally difficult to eliminate through subsequent curing treatment [15]. This seriously restricts the mechanical properties and structural load-bearing capacity of the product [16,17,18]. Therefore, it is of great significance to understand and control the fiber arrangement, interlayer slip and local strain distribution in the preforming stage from the perspective of microstructure to achieve defect suppression and improve the quality of material preforming.

Regarding the deformation mechanism and process control of the preforming process, scholars have conducted a series of studies, analyzing the forming behavior of pre-impregnated materials from different perspectives such as theoretical modeling, device design, and numerical simulation. For instance, Qi Junwei et al. [19] established a theoretical analysis model for preforming based on a C-shaped long beam and designed a preforming bending test device, laying the foundation for understanding the deformation mechanism characteristics of pre-impregnated materials during the continuous forming process. Chen Yichuan et al. [20,21] designed preforming devices by constructing a series of deformation constraint surfaces. Gao Shaonan et al. [22] corrected the original theoretical model based on a tension control preforming scheme, enabling the pre-impregnated materials to closely adhere to the mold surface. Chen Boyi et al. [23] optimized and reconstructed the mold surface based on the ABAQUS simulation results, thereby improving the adhesion between the pre-impregnated materials and the preforming mold. However, the existing research mainly focuses on open or semi-open mold systems, which have limited constraint ability on material deformation and are difficult to achieve effective control of the complex deformation behavior during the preforming process. Moreover, the related process parameter studies mostly remain at the empirical level, lacking systematic analysis of the parameter action mechanism and its relationship with defect formation. Finally, existing studies mainly focus on the macroscopic molding quality or mechanical properties, and do not systematically analyze the effects of microstructure such as fiber arrangement, interlayer contact state, and local deformation, which limits the in-depth understanding of the defect formation mechanism.

In addition, although there is still a relatively limited systematic research on the advanced pultrusion preform process, relevant studies from other composite material forming processes (such as thermal diaphragm [24,25], compression molding [26,27,28], and traditional pultrusion [29,30]) can provide certain references. Among them, the inappropriate mold surface and process parameters are the main reasons affecting the formation of defects in the prepreg during the preform process. The mold surface changes the geometric constraints of the prepreg, directly influencing its deformation path and stress state, thereby inducing the formation of defects. For example, Azzouz et al. [31] found that the material is prone to generate local strain at positions with intense geometric changes, which easily leads to the generation of wrinkles and other defects; Viisainen et al. [32] found that the severity of wrinkles largely depends on the mold curvature, the larger the cone angle and Gaussian curvature of the convex mold surface, the lighter the wrinkles produced; meanwhile, the outward-bending mold surface usually produces less wrinkles than those with zero curvature or inward bending. In addition, the setting of process parameters also has a significant impact on the generation of defects. For instance, Farnand K et al. [33] found that internal wrinkling defects occurred in the prepreg at 0° and 90° layers under low temperature; Erland et al. [34] found that increasing the heating temperature of the un-cured prepreg would first reduce and then increase the critical shear stress between layers. Vedernikov et al. [35] found that the difference in pultrusion speed affects the shape deformation, microstructure and mechanical properties of the composite material, and a pultrusion speed of 10 mm/s would result in obvious delamination. Moreover, although the research on the influence of preform tension on material deformation and defect evolution is still relatively limited, Zhao Cong et al. [36] through laying experiments found that excessive or insufficient tension would reduce the laying quality; Zhang Shijie et al. [37] speculated that the influence of winding tension on carbon fiber winding components is the combined result of three factors: fiber wear, fiber volume fraction change and prestress.

In recent years, nanomodified prepreg and hybrid prepreg systems have received much attention in the field of composites. Studies have shown that the introduction of nano-fillers into prepreg can improve interlayer properties, but also bring process challenges in terms of dispersion uniformity, resin fluidity and curing kinetics changes [38,39]. Similarly, hybrid prepreg tapes generally outperform conventional roving in terms of mechanical properties, providing new material options for continuous forming processes [40]. However, the current research on these advanced material systems mainly focuses on the preparation and performance research stages [41,42,43], and the systematic research on their application in continuous molding processes such as advanced pultrusion (ADP) is still very limited. The die surface optimization method and process parameter analysis method proposed in this paper provide a theoretical basis and method support for the application of this new prepreg system in continuous molding in the future.

Therefore, this paper takes L-shaped asymmetric composite material components as the research object and conducts research on the preforming mold surface optimization and process parameter analysis for defect suppression. Firstly, a mold surface optimization method based on three-dimensional strain homogenization is proposed. By controlling the strain distribution in the in-plane and thickness directions, the fiber arrangement becomes more uniform and the interlayer slip is controllable, thereby suppressing the formation trend of defects such as wrinkles, uneven thickness and local fiber buckling. Subsequently, an analysis of the influence of process parameters (temperature, traction speed, tension) on the preforming quality was carried out on the optimized mold. The related research can provide a theoretical basis for the design of preforming molds and process optimization in advanced pultrusion forming processes.

## 2. Materials, Equipment and Methods

### 2.1. Materials

A unidirectional carbon fiber/epoxy prepreg (EH919-34–12KHF40C-U-194 gsm) supplied by Jiangsu Hengshen Co., Ltd. (Danyang, China) was used in this study. The thickness of a single ply before curing was 0.19 mm, and its detailed properties are listed in Table S1. A polytetrafluoroethylene (PTFE) high-temperature release fabric, with a thickness of 0.08 mm, was procured from a commercial PTFE tape supplier (Taizhou, China).

### 2.2. Construction of the Simulation Model

ABAQUS 2021 was used to construct and analyze the finite element simulation model. Due to the significant performance differences in the fibers in the unidirectional prepreg of composite materials in the longitudinal and transverse directions, the prepreg exhibits obvious directionality [44]. Relevant studies have shown that prepregs are approximately uniform in the transverse direction. Therefore, most of the related simulations use transverse anisotropic constitutive models to characterize the mechanical behavior of prepregs [45,46]. The corresponding material parameters are provided in Section 2.1. During the preforming process, the mold temperature remains constant and is relatively low compared to the curing temperature. The prepreg was therefore assumed to undergo an isothermal process, with the evolution of the degree of cure neglected [44]. A general contact formulation was employed in the simulation. The normal contact behavior was defined as hard contact, while the tangential behavior followed a penalty-based friction formulation. In the actual manufacturing process, PTFE release material was applied to the surface of the prepreg, and a Teflon coating was sprayed on the mold surface, thereby significantly reducing the friction that the prepreg experienced during actual molding. To simplify the model and highlight the influence of the mold shape on the preforming behavior, this paper does not explicitly model the isolation film. Instead, the friction coefficient is set to 0.1 to simulate the friction behavior during the preforming process under the action of the isolation film and the coating.

To replicate the actual preforming process, the simulation was divided into two steps. In the first step (mold closing), the lower die was fixed, while the upper die was driven downward under displacement control to achieve the initial forming of the prepreg. During this stage, a dynamic explicit analysis was employed, and the analysis time was set at 0.1 s. The second step is the traction forming stage, the upper and lower molds are completely fixed, and the traction motion of displacement control is applied at the front end of the prepreg along the forming direction, so that the material moves forward under the mold constraints and completes the preforming process. The analysis time for this stage is set at 160 s, and the pulling speed is 0.5 mm/s to ensure that the pulling process has a quasi-static characteristic. To improve the calculation efficiency, the mass scaling method is adopted under the condition of ensuring quasi-static deformation, and the mass scaling factor is set to 10,000. To ensure the validity of the analysis results, the ratio of kinetic energy (KE) to internal energy (IE) of the system is monitored in real time during the calculation process. The results showed that KE/IE remained below 5% throughout the forming process, satisfying the quasi-static condition. Furthermore, to simulate the tension that the prepreg material experiences during the actual preforming process, a constant tension load was applied to the front and rear ends of the prepreg material in both stages of the entire preforming analysis. The tension magnitude was set at 40 N, ensuring that the prepreg material remained straight during the forming process. The specific boundary condition diagram is shown in Figure S1.

The preforming mold was set as a rigid body, and hexagonal three-dimensional solid elements (C3D8) were used for meshing. The meshes are relatively loose to improve the computational efficiency. The prepreg, as the main deformed area in the preforming process, is meshed with finer grids and densely distributed in key deformed areas to ensure the calculation accuracy of these key deformed areas [47]. Based on the deformation process and material properties of the prepreg during the preforming process, single-precision dense seed distribution was carried out on the prepreg parallel to and perpendicular to the pultrusion direction, and hexagonal continuous shell elements SC8R were used for grid division. The thickness of the prepreg is defined by the nominal thickness, and three integral points are set in the thickness direction to reasonably reflect the stress distribution characteristics in the thickness direction. Taking the initial mold as an example, the detailed mesh configuration is shown in Figure S2.

### 2.3. Experimental Setup and Forming Procedure

**Figure 1:**
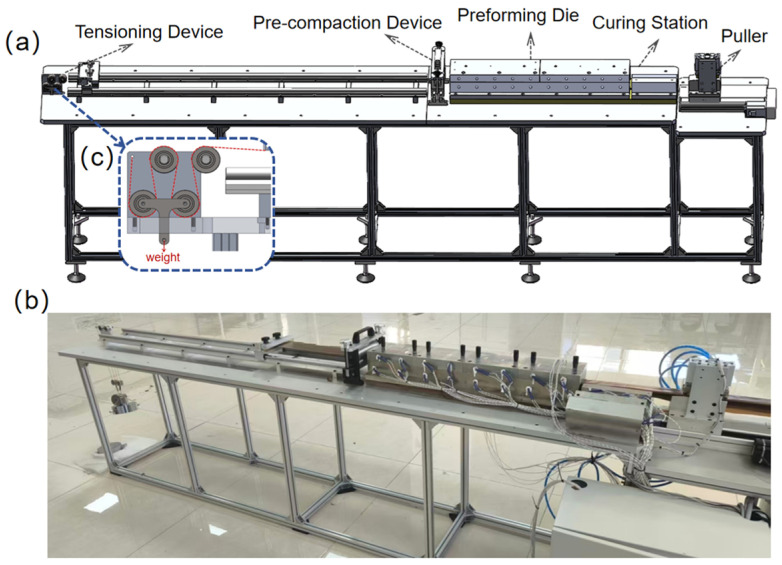
Preforming experimental system: (**a**) design schematic, (**b**) photograph, and (**c**) detail of the tension weight configuration. The red dotted line in (**c**) represents the cable routing between the pulleys in the tension application setup.

The experimental setup used in this study is a small-scale preforming experimental system that was designed and constructed independently. This system mainly consists of a preforming mold, a curing mold, a pulling device, a tensioning device and a preliminary compaction device, and can adjust key parameters such as temperature, pulling speed and tension. The temperature is monitored and controlled by electric heating tubes and platinum resistance temperature sensors. Before the experiment, the mold surface temperature was calibrated, and the results showed that the temperature difference could be controlled within ±2 °C, which met the stability requirements of the preforming process. Additionally, the tension is achieved by configuring replaceable weights below the double pulley mechanism. The tension force exerted on the material during the experiment is half of the total weight of the hanging pulley and the weights below (as shown in Figure 1c). It is worth noting that the curing mold in this study consists only of an open rigid lower mold, without an upper mold, and no external pressure is applied. This is because the curing process in this study is not intended to replicate the final pultrusion curing process, but rather to “freeze” the microstructure state after preforming, so as to conduct subsequent research on internal quality. A schematic illustration and photograph of the experimental setup are shown in Figure 1.

The pre-impregnated materials were stored in a −18 °C freezer and were taken out 4 h in advance for natural thawing. Before the specific experiment began, the shapes were drawn using CAD 2018 and the pre-impregnated material strips of different angles were cut using a cutting machine. During the experiment, the manual layup was carried out according to the target layup sequence, and the initial compaction was performed to reduce the interlayer gaps. Then, the mold was opened and the pre-impregnated materials covered with the release fabric were successively passed through each device, and the material was clamped and tensioned by the traction device and the tension device. After the material positioning was completed, the preforming mold was closed and each mold was heated to the set temperature. Then, the traction device was started to achieve the continuous preforming process at the set speed.

### 2.4. Experimental Design

The core process parameters of the advanced pultrusion preforming process mainly include temperature, pulling speed, tension, etc. Among them, the selection of the preforming temperature needs to take into account both the material’s curing behavior and rheological properties. In the actual process, the temperature during the preforming stage is usually kept constant and should be significantly lower than the resin’s initial curing temperature to avoid premature reaction. By studying the dynamic DSC test results under different heating rates and performing linear fitting on the exothermic peak characteristic parameters, the curing starting temperature of the prepreg is approximately 160 °C (as shown in Figure S3a,b). Therefore, the preforming process needs to be carried out under a condition much lower than this temperature.

Furthermore, the preforming temperature mainly affects the deformation and flow behavior of the material by regulating the resin viscosity: when the viscosity is high, inter-layer slip is restricted, which may lead to local wrinkling or inconsistent deformation; meanwhile, a low viscosity may cause excessive resin flow, weakening the structural stability. Relevant studies have found that when the resin viscosity is between 10 and 700 Pa·s, a reasonable balance can be achieved between the flowability and structural stability, thus meeting the basic requirements of the preforming process for fluidity and conformality [48,49]. Combined with the resin viscosity–temperature curve of the prepreg in this paper (as shown in Figure S3c), the appropriate temperature range for the preforming stage is preliminarily determined to be 60–100 °C. Therefore, in this experiment, 60 °C, 70 °C, 80 °C, 90 °C and 100 °C were selected as the experimental temperatures.

Meanwhile, the pulling speed mainly affects the preforming process by altering the deformation rate of the material in the mold. A too low pulling speed will significantly reduce the processing efficiency, thereby increasing the overall cost of molding; meanwhile, a high pulling speed causes the pre-impregnated material to deform at a higher rate, and the interlayer slip and fiber rearrangement cannot be coordinated in time, resulting in defects. As Vedernikov et al. [35] discovered, when the speed reaches 10 mm/s, the material will exhibit obvious delamination defects. Therefore, the pulling speeds of 0.1 mm/s, 0.3 mm/s, 1 mm/s, 3 mm/s, 6 mm/s, and 10 mm/s were selected. The preliminary test results showed that the preforming quality was relatively better when the pulling speed was between 0.3 and 1 mm/s. Further experiments on intermediate speed levels were also conducted. In addition, the tension regulates the force level of the pre-impregnated material in the molding direction and the overall tension degree, influencing the fiber deformation path and interlayer coordinated slip behavior. When the tension is low, the overall tension degree of the material is low, and the interlayer interaction mainly relies on the adhesive effect of the resin, resulting in local instability; meanwhile, a too high tension will cause the material to be in an overly tight state, thereby inhibiting its deformation ability.

Based on the above analysis, the study adopted a single-factor experimental method to separately analyze the influence patterns of the preforming temperature, pulling speed, and tension. The specific experimental design is shown in Table 1.

It should be noted that this study adopts a single-factor experimental design to preliminarily explore the influence of various process parameters on the quality of preforming. This method can clearly demonstrate the influence trend of each parameter on the preform quality under a limited experimental volume, but it cannot systematically reveal the interaction among the parameters, and its results still have certain limitations. Future work will further explore these coupling effects through a multifactorial design.

### 2.5. Characterization of Preforming Defects and Evaluation Metrics

The total forming length of the preformed part is approximately 2 m. To facilitate comparative analysis, a 300 mm area with stable forming in the middle of the sample was selected as the research object to study its appearance quality, thickness uniformity and internal quality. Among them, the appearance quality mainly observes whether there are obvious defects such as scratches, gelomas, depressions, and surface wrinkles on the surface of the preformed body.

The uniformity of thickness is quantified by the variance of thickness samples. The study area is divided into three regions along the forming direction: the front, middle, and rear. Measurement points are arranged at the center of the web at key sections, the inner arc zone at the corners, the outer arc zone at the corners, and the center of the edge strip in each of these regions, thereby achieving a comprehensive assessment of the thickness variations in different structural areas [50]. To eliminate the influence of uneven regional deformation on the statistical results, the variance of thickness samples for each region was calculated separately, and the average of these regional variances was used as the overall indicator of thickness uniformity. The distribution of thickness measurement points is shown in Figure S3.

The internal quality is characterized by preparing metallographic samples and conducting microscopic observation. Considering that the pre-formed samples have not yet solidified, they need to be heated at 195 °C in a curing mold for 1.5 h for curing, thus ensuring the integrity of sample preparation. The cross-section of the middle area is selected as the microscopic observation surface. Metallographic samples are prepared through inlaying and polishing processes, and the metallographic samples are observed using an ultra-depth microscope.

## 3. Mold Surface Design

### 3.1. Initial Mold Scheme

#### 3.1.1. Geometric Model and Initial Mold Design

This paper selects a typical composite L-shaped beam component as the research object. The target cross-sectional dimensions are 31.455 mm × 32.844 mm, and the inner angle radius is 4 mm. This type of structure is widely used for connection and load transfer between different structural units. Due to the presence of obvious corner areas in this type of component, the fibers need to undergo significant path bending and spatial rearrangement during the preforming process, which easily leads to various defects in the corner areas and thus has typical research significance.

During the actual forming process, considering factors such as the deviation of the pre-impregnated material placement and processing errors, an additional 10 mm of coating margin was added on both sides of the L-shaped beam section to ensure that the dimensions after trimming still meet the design requirements; the process extension area was further increased by an additional 15 mm to provide space for material offset and resin flow. Based on the above structure, a geometric model of the preforming process as shown in Figure S4 was established. Here, line segment A_1_B_1_C_1_D_1_ represents the initial pre-impregnated material section before deformation, curve ABCD represents the target section after deformation, and curve ABCD consists of line segment AB, line segment CD, and a 1/4 circle with a radius of 4 mm, BC. To further describe the deformation process of the material, a spatial coordinate system is established with the material forming direction as the z-axis and the plane where the forming section ABCD is located as the xy-plane. The z-axis coordinate of the initial cross-section A_1_B_1_C_1_D_1_ of the prepreg is set as Z_1_ (the preforming mold length), and the y-axis coordinate is set as Y_1_ (the initial placement height). At this time, the coordinates of each point are as follows:

A (0,57.844,0), B (52.455,57.844,0), C (56.455,53.844,0), D (56.455,0,0), A_1_ (0,Y_1_,Z_1_), B_1_ (52.455,Y_1_,Z_1_), C_1_ (58.738,Y_1_,Z_1_), D_1_ (112.582,Y_1_,Z_1_).

During the preforming process, the structural parameters of the mold surface directly affect the deformation path and stress distribution of the material. Among them, the discharge height Y_1_ determines the spatial position of the prepreg entering the mold surface from the inlet section, thereby influencing the spatial deformation path of the fibers on the mold surface; the mold length Z_1_ determines the progressive deformation distance of the prepreg in the preforming mold, thereby affecting the intensity of the spatial deformation of the fibers on the mold surface. Based on the actual equipment conditions and engineering experience, Y_1_ = 55 mm and Z_1_ = 800 mm were selected as the initial mold surface structure parameters, and the initial mold surface was constructed accordingly.

Different from the traditional open preforming molds, this paper adopts a closed preforming mold structure with the collaborative action of the upper and lower molds to enhance the constraint ability and forming stability of the preforming process. This paper focuses on the optimization design of the mold surface and does not involve the comparative analysis of different mold structure forms. The distance between the upper and lower molds of the preforming mold is determined by the material thickness and process requirements: the thickness of the single layer of the pre-impregnated material used in the experiment is 0.19 mm, with a total of 16 layers. Considering a 0.08 mm layer of polytetrafluoroethylene release fabric, the total thickness is approximately 3.2 mm. However, after multiple compression measurements, the actual thickness of the experimental material in the free state is approximately 3.3 mm, slightly higher than the theoretical calculation value. Since the preforming stage aims mainly at preliminary shaping, a too small mold gap would significantly increase the feeding resistance of the material, and thus 3.4 mm was selected as the distance between the upper and lower molds, and accordingly the final preforming mold structure was determined.

#### 3.1.2. Defect Analysis Under the Initial Mold Design Scheme

Based on the actual preforming process, we abandon the rapidly deformed prepreg that was already inside the mold during the mold closing stage, and focus on analyzing and studying the new prepreg that enters the mold after the mold closing. The deformation states of this part of the prepreg at different positions within the mold can be approximately regarded as the deformation states in each stage of the preforming process.

**Figure 2:**
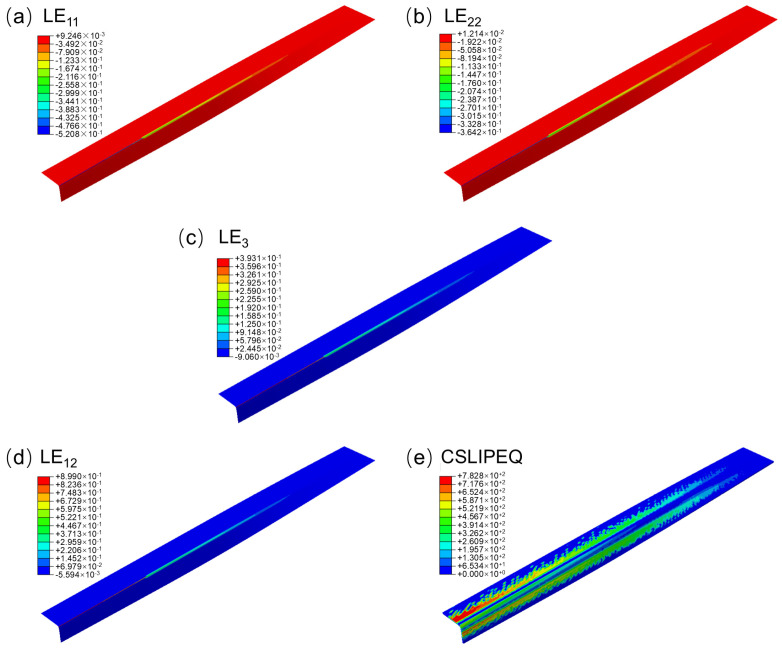
Contour plots of (**a**) LE_11_, (**b**) LE_22_, (**c**) LE_33_, (**d**) LE_12_, and (**e**) CSLIPEQ distributions.

The logarithmic strain maps in different directions during the preforming process under the initial mold constraints are shown in Figure 2a–c. The logarithmic strains LE_11_ parallel to the fiber direction, LE_22_ perpendicular to the fiber direction, and LE_33_ in the thickness direction all exhibited significant abrupt changes in the corner region, with rapid strain variations and the formation of local concentrated areas. Specifically, the maximum positive logarithmic strain LE_11_ and LE_22_ during the preforming process were relatively small, while the maximum negative logarithmic strain was relatively large, reaching 52.08% and 36.24% respectively. Moreover, the maximum negative logarithmic strain of both was mainly located in the corner region, indicating that the prepreg underwent compressive deformation in the fiber direction and perpendicular to the fiber direction in this region. In contrast, the maximum positive logarithmic strain LE33 was relatively large, reaching 39.31% and also occurring in the corner region, while the maximum negative logarithmic strain was relatively small. This indicates that the prepreg alleviated local deformation by releasing in the thickness direction under the constraint of in-plane compression.

In conclusion, LE_11_, LE_22_ and LE_33_ show opposite change trends during the preforming stage, indicating that the deformation behavior of the prepreg within the initial profile mold is poorly coordinated in the planar and thickness directions. This might be due to the fact that the geometric shape changes in the corner area are more intense, while the transition of the initial mold profile is relatively straightforward, making it difficult for the material in this area to achieve shape reconstruction through progressive deformation. As a result, deformation is concentratedly released or accumulated in local areas. The aforementioned sudden strain changes and insufficient strain direction coordination can, on one hand, cause a sudden transition in the stress state of the material within the mold, and on the other hand, lead to a sharp increase in the strain gradient, thereby increasing the tendency of local areas to develop preforming defects such as in-plane buckling and wrinkling.

The distribution cloud map of the logarithmic shear strain LE_12_ is shown in Figure 2d. During the preforming stage, LE_12_ also shows an uneven distribution and a significant shear strain appears in the corner area. This indicates that the prepreg mainly achieves geometric deformation through in-plane shear deformation in the corner area. However, excessive in-plane shear may cause fiber orientation deviation, thereby weakening the stability of the material in the compression direction and increasing the risk of local buckling and wrinkling.

Further, by combining the equivalent slip quantity CSLIPEQ as shown in Figure 2e, it can be seen that the CSLIPEQ values in the center regions of the web and flange near the L-shaped section end are relatively large and change rapidly. Further, by combining the equivalent slip quantity CSLIPEQ (Equivalent Contact Slip, a scalar measure of the cumulative tangential relative motion between contacting surfaces), as shown in Figure 2e, it can be seen that the CSLIPEQ values in the center regions of the web and flange near the L-shaped section end are relatively large and change rapidly. This indicates that the prepreg releases deformation energy through inter-laminar or inter-fiber slip in this area to achieve geometric coordination. However, the equivalent slip quantity in the corner area is relatively small, indicating that the deformation release capacity in this area is limited. When the in-plane compression strain accumulates continuously and cannot be effectively released or alleviated through slip, it is prone to cause compression strain concentration and local instability, thereby promoting the formation of wrinkles and other defects.

By integrating the deformation characteristics of LE_11_, LE_22_ and LE_33_, and combining with the distribution of LE_12_ and CSLIPEQ, it can be seen that the initial mold design can achieve the overall preforming of the prepreg. However, in the corner areas, there are obvious in-plane compression, thickness release and shear slip deformations, which lead to local strain concentration and insufficient deformation coordination. Although this problem did not result in deformation failure, it is the main cause of wrinkles, fiber buckling and size deviations and other defects. Therefore, it is necessary to further optimize the mold surface to improve the problems of local strain concentration and insufficient deformation coordination, and thereby suppress the occurrence of related defects.

### 3.2. Optimization Design of Mold Surface

From the aforementioned analysis, it can be concluded that under the initial mold surface conditions, the pre-impregnated material exhibited significant strain concentration and deformation inconsistency during the preforming process, especially in the L-shaped cross-section corner area and the region near the end of the molding. This local deformation imbalance is prone to cause typical defects such as fiber wrinkling and accumulation, indicating that the material’s spatial deformation capability under the initial mold surface has not yet met its deformation requirements.

Once the structural parameters such as the discharge height, mold length and the spacing between the upper and lower molds are changed, the boundary conditions of the material and the deformation mode will also change simultaneously, thereby increasing the complexity of the analysis and weakening the independence of the profile factors. Therefore, in order to highlight the influence of the mold profile on the material’s deformation behavior, this paper, while keeping the above structural parameters unchanged, only optimizes the profile of the intermediate transition area of the mold, so that the material can achieve progressive deformation during the transition from a flat surface to the target cross-section, reducing local strain concentration and improving deformation coordination. To quantitatively evaluate the optimization effect of the profile, the amplitudes of logarithmic strain LE_11_, LE_22_ and LE_33_ are selected as the main evaluation indicators, which are controlled within ±5%, and the uniformity of strain distribution is used as an auxiliary criterion. Thus, a mold profile optimization method with the goal of uniform three-dimensional logarithmic strain distribution is proposed.

Specifically, several characteristic cross-sections are selected along the preforming direction to represent the geometric change process of the mold surface. Considering that the pre-impregnated material undergoes the most severe deformation in the region close to the L-shaped cross-section end, and the deformation is relatively gentle in the region close to the plane entrance, this optimization design arranges the characteristic cross-sections densely in the region close to the L-shaped cross-section end. Cross-sections are taken at distances of 50 mm, 100 mm, 200 mm, 400 mm, and 700 mm from the L-shaped cross-section end and the key nodes on the cross-sections are extracted. Among them, the cross-sections at 50 mm and 100 mm are used to adjust the severe deformation near the L-shaped corner area, the cross-sections at 200 mm and 400 mm are used to achieve a smooth transition of deformation, and the cross-section at 700 mm is used to coordinate the deformation in the plane entrance area, thereby ensuring the continuity and uniformity of the entire surface change.

**Figure 3:**
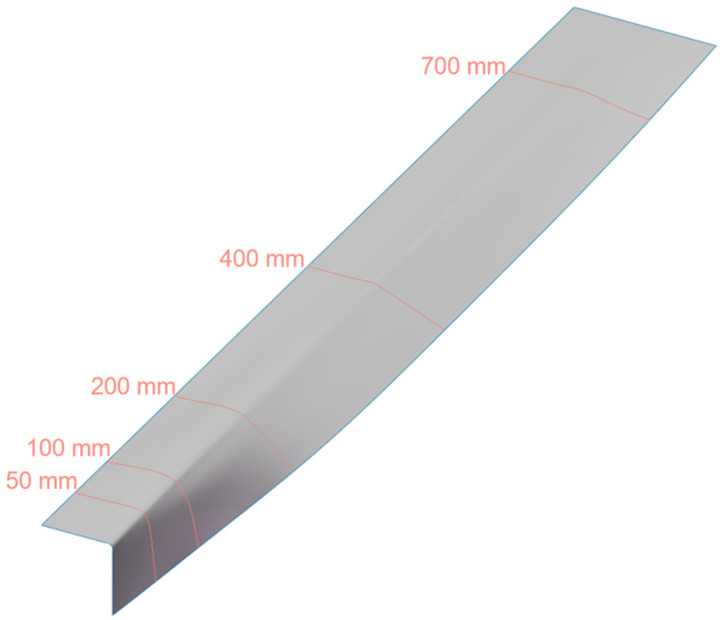
Optimized profile diagram of preformed mold.

Based on this, by adjusting the curvature of the corner areas and the transitional forms in each feature section, the initial mold profile was reconstructed, and the above optimization process was repeated based on the modified sections. Through multiple rounds of “surface adjustment–simulation analysis–result feedback” closed-loop iterations, the shape of the section curves was continuously corrected until the positive and negative amplitudes of LE_11_, LE_22_, and LE_33_ were all controlled within ±5%. The final optimized mold profile schematic diagram is shown in Figure 3. Compared with the initial mold profile, this profile has a more gentle deformation in the corner area, which makes the deformation of the prepreg in all directions more coordinated and uniform, and effectively suppresses the occurrence of typical defects.

## 4. Results and Discussion

### 4.1. Optimization Effect of Mold Surface Design

#### 4.1.1. Comparison Before and After Optimization

**Figure 4:**
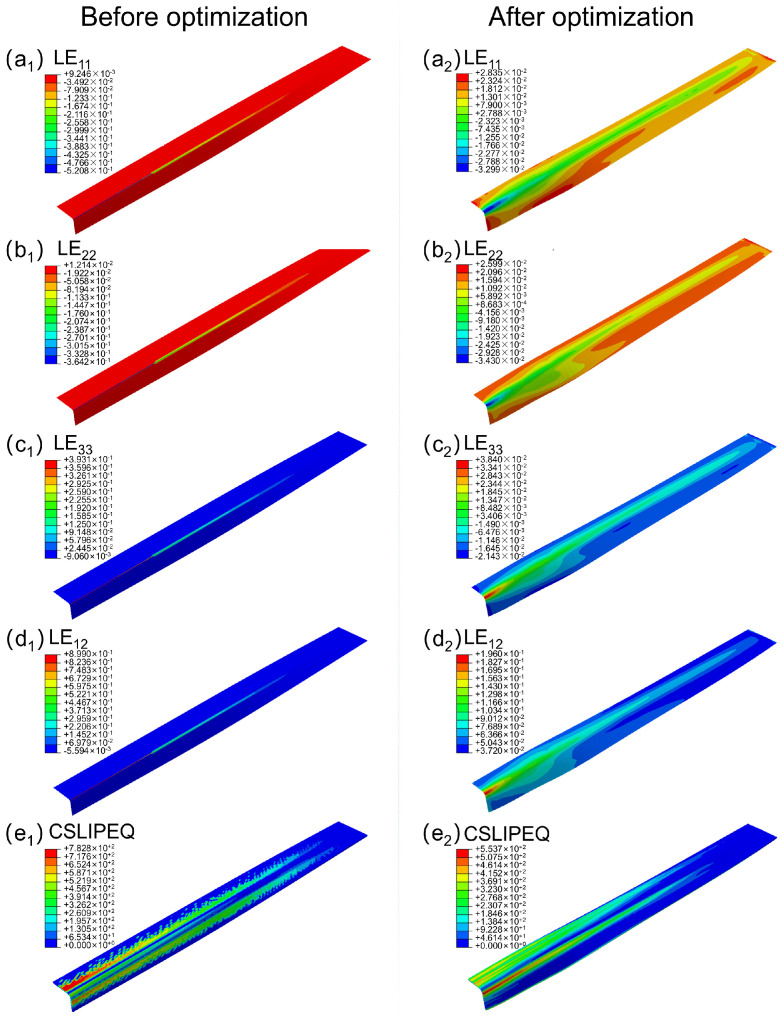
Comparison of cloud images before and after optimization. Left column (**a_1_**–**e_1_**): Before optimization. Right column (**a_2_**–**e_2_**): After optimization. Each row corresponds to (**a**) LE_11_, (**b**) LE_22_, (**c**) LE_33_, (**d**) LE_12_, (**e**) CSLIPEQ.

The comparison cloud charts of the preforming deformations of LE_11_, LE_22_ and LE_33_ before and after optimization are shown in Figure 4a–c. Overall, compared with the preforming deformations under the initial profile mold, the preforming deformations after profile optimization are more gentle and uniform. Compared with the local strain concentration (the maximum strain reached 52.08%) in the corner area under the initial mold, the maximum positive and negative strain values in all directions after optimization are all controlled within 5%, indicating that the material deformation has gradually changed from local concentration to a continuous deformation process along the forming path.

From the perspective of the in-plane logarithmic strain distribution, the maximum negative logarithmic strain of LE_11_ decreased from 52.08% to 3.23%, while that of LE_22_ decreased from 52.08% to 3.43%. This indicates that the material has shifted from the original dominant in-plane compression deformation to a more balanced tensile-compressive co-operative deformation mode. In contrast, the maximum positive logarithmic strain of LE_33_ decreased from 39.31% to 3.84%, showing an opposite trend. This suggests that under the optimized surface mold, the material no longer exhibits the obvious thickness release area caused by the concentrated in-plane compression within the initial mold surface shape, but instead gradually adjusts the thickness deformation state along the forming path in a more gradual manner, making the distribution of tensile and compressive deformations in the thickness direction more uniform.

Consequently, the comparison cloud map of the pre-formed deformation of LE_12_ before and after optimization is shown in Figure 4d. The maximum positive strain of the optimized LE_12_ has significantly decreased, and the overall shear strain distribution has shifted from being concentrated in the corner area to a gradually transitioning uniform distribution along the forming direction. This change indicates that the material no longer relies on local strong shear deformation to achieve shape transition in the geometric turning area, but instead completes the structural forming through more uniform in-plane deformation gradually, thereby effectively alleviating the problem of local shear concentration.

Further analysis was conducted by combining and optimizing the pre-formed deformation of CSLIPEQ before and after the process. As shown in Figure 4e, the maximum value of the CSLIPEQ after the profile optimization also decreased. The abrupt change phenomenon that was originally near the end web and edge strip center of the L-shaped section has basically disappeared. The slip distribution shows a more continuous and gentle changing trend. This indicates that the contact state of the material in the optimized profile mold is more stable, and the interlayer relative slip behavior has been effectively mitigated, thereby avoiding problems such as material accumulation or interlayer misalignment caused by excessive local slip.

In conclusion, the optimized design of the mold surface significantly improves the deformation coordination and uniformity of the material during the preforming process, making the shear and slip deformations more stable. This, in turn, helps to suppress the tendency of defects such as corner wrinkles and in-plane buckling, and significantly improves the dimensional consistency of the preformed parts, providing a more stable structural foundation for subsequent preform experimental verification and process parameter research.

#### 4.1.2. Sample Production

To further verify the effect of surface optimization, a preforming part trial was conducted for the target layup. The preforming temperature was set at 80 °C, the pulling speed was 0.3 mm/s, and the tension was 30 N. The appearance quality of the preforming part showed no obvious defects such as scratches, lumps, depressions, or surface wrinkles. The sample only had extremely slight local unevenness, and the overall surface uniformity was good. Additionally, the sample variance of the thickness was 1.16 × 10^−4^ mm^2^. The internal quality was overall structurally complete, and no obvious fiber wrinkles, macroscopic layering, or through-type defects were observed. Therefore, it is believed that the target layup can meet the basic forming requirements under the current process conditions. The specific result photos can be found in Section 4.2.1.

**Figure 5:**
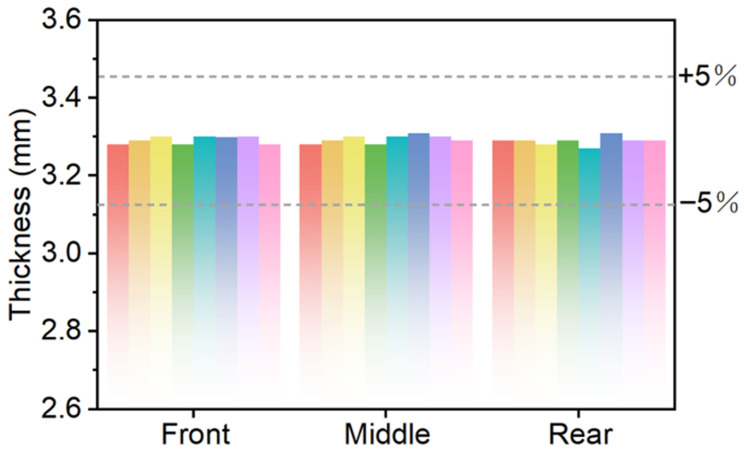
Experimental results of thickness measurement under the target layup. Different colors in the figure represent the thickness values at various cross-sectional positions.

Furthermore, in order to facilitate comparison with the simulation results for verifying the correctness of the simulation results, the logarithmic strain requirement in the thickness direction (|LE33| < 5%) in the simulation was converted into a thickness evaluation index; that is, the thickness deviation was controlled within the range of the reference thickness ±5%. Considering that the actual thickness of the prepreg usually deviates from the theoretical layup thickness, the average thickness of all measurement points was used as the reference thickness. The study found that the thickness at each measurement point was within the range of the reference thickness ±5%, and no abnormal points exceeding the range were found. The overall thickness distribution was relatively uniform (as shown in Figure 5). By comparing the simulation results, it can be found that the thickness distribution trend measured experimentally is in good agreement with the thickness strain distribution in the simulation. This indicates that the established finite element model can accurately predict the thickness change pattern during the preforming process, and the optimized mold surface has good effectiveness in improving the uniformity of deformation.

### 4.2. Effect of Process Parameters

#### 4.2.1. Effect of Preforming Temperature

**Figure 6:**
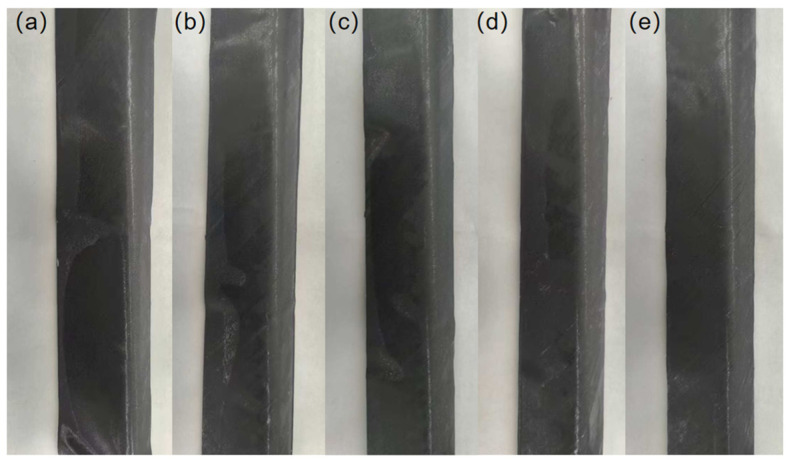
Photos of appearance quality at different preforming temperatures: (**a**) 60 °C, (**b**) 70 °C, (**c**) 80 °C, (**d**) 90 °C, (**e**) 100 °C.

The influence of preforming temperature on the appearance quality is shown in Figure 6. Within the study range, the appearance quality showed a trend of “improvement first, then deterioration” as the preforming temperature increased. As shown in Figure 6a, when the preforming temperature was low (60 °C), the sample had obvious waves and depressions, and the surface flatness and uniformity were extremely poor. With the increase in the preforming temperature (70–90 °C), as shown in Figure 6b–d, the surface flatness and uniformity of the samples are significantly improved. The samples at 70 °C still have slight local rippling and uneven areas on the surface, while the samples at 80 °C and 90 °C only have very slight local unevenness and no obvious surface defects such as rippling and depression. The surface has good smoothness and uniformity. However, as the temperature further increases (100 °C), as shown in Figure 6e, the surface flatness and uniformity of the sample decrease, but the overall difference is not significant compared to 80 °C and 90 °C.

**Figure 7:**
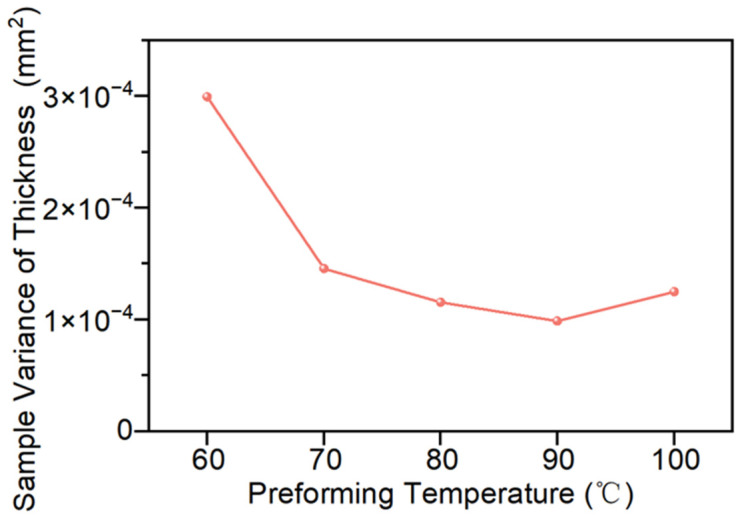
Thickness sample variances at different preforming temperatures.

The influence of preforming temperature on thickness uniformity is shown in Figure 7. Within the study range, the sample variance of thickness dimensions decreases first and then increases as the preforming temperature rises. When the preforming temperature increases from 60 °C to 90 °C, the sample variance of thickness decreases from 2.99 × 10^−4^ mm^2^ to 9.88 × 10^−5^ mm^2^, with a reduction of approximately 67%, indicating that the thickness dispersion significantly decreases and the overall uniformity significantly improves. However, when the temperature continues to increase to 100 °C, as shown in Figure 7, the overall variance of thickness increases to 1.25 × 10^−4^ mm^2^.

**Figure 8:**
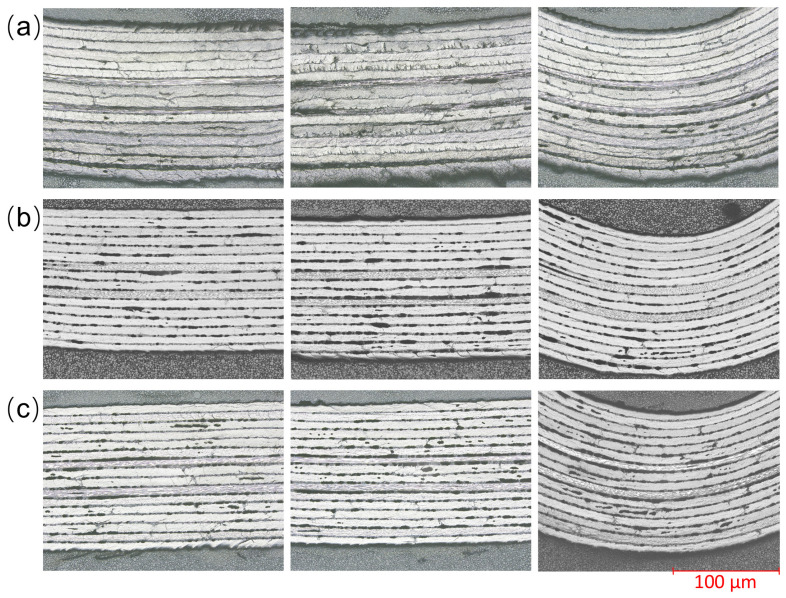
Metallographic microstructure photos at different preforming temperatures: (**a**) 80 °C, (**b**) 90 °C, (**c**) 100 °C.

The influence of preforming temperature on internal quality is shown in Figure 8. The experiments only analyzed the specimens formed under the pretemperature conditions with better appearance quality and thickness uniformity (80, 90, 100 °C). As shown in Figure 8a, when the preforming temperature is 80 °C, the interlayer interfaces within the laminate are blurred and have poor continuity. Some wavy fiber undulations can be observed to a certain extent, and micro-crack defects can be seen in some local areas. As shown in Figure 8b,c, when the preforming temperature increases to 90 °C and 100 °C, the interlayer interfaces within the laminate become clearer and more distinct, and the wavy fiber undulations are reduced. In addition, some obvious dark areas could be seen inside each sample, which might be pseudo-defects caused by grinding and polishing during the metallographic sample preparation, or related to local small pores. Considering that the research object of this paper is the preforming stage, such defects can usually be effectively suppressed in the subsequent curing and forming process, and thus will not be further discussed.

This changing trend is closely related to the variation in resin viscosity: when the preforming temperature is relatively low, the resin viscosity is high, the interlayer slip is restricted, and the material is prone to local instability during deformation, thereby resulting in appearance defects, uneven thickness and internal defects. However, when the temperature gradually rises to 90 °C, the resin viscosity gradually decreases to an appropriate range. The resin can flow effectively and provide a lubricating effect, promoting the relative slip and redistribution of the fiber bundles [51], thereby gradually achieving the best comprehensive quality of the preform. When the preforming temperature is too high, the low resin viscosity will induce obvious resin migration behavior, forming local regions with high and low resin concentrations, thereby reducing the uniformity of the pre-form thickness.

In conclusion, within the scope of this study, the influence of preforming temperature on preforming quality shows a trend of “improvement first, then deterioration”. Among them, when the preforming temperature is 90–100 °C, the appearance quality and internal structure of the sample are relatively stable with no significant difference, indicating that this range is a relatively reasonable preforming temperature range. However, the thickness uniformity is best at 90 °C, so it shows a relatively optimal preform quality when considering all quality indicators.

#### 4.2.2. Effect of Pulling Speed

**Figure 9:**
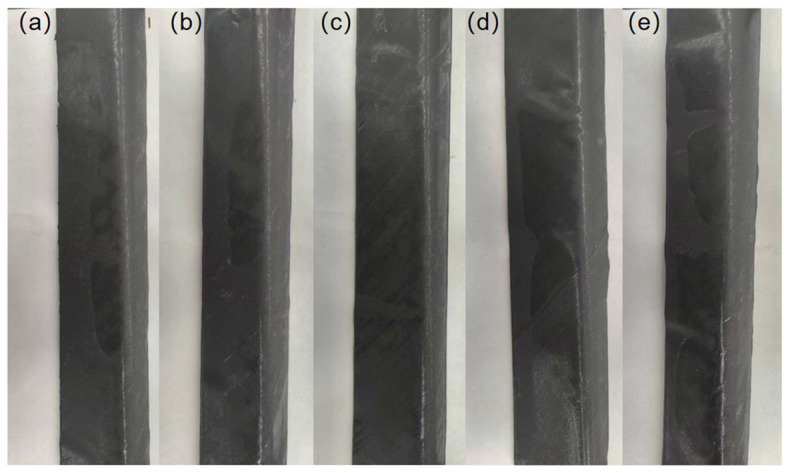
Photos of appearance quality at different pulling speeds: (**a**) 0.1 mm/s, (**b**) 0.3 mm/s, (**c**) 0.6 mm/s, (**d**) 1 mm/s, (**e**) 10 mm/s.

The influence of pulling speed on the appearance quality is shown in Figure 9. Five representative sample research areas were selected at pulling speeds of 0.1, 0.3, 0.6, 1, and 10 mm/s to compare the appearance. Within the study range, the appearance quality shows a trend of “improvement first, then deterioration” as the pulling speed increases. As shown in Figure 9a, when the pulling speed is slow (0.1 mm/s), obvious irregular undulations appear on the sample surface, and the surface flatness and uniformity are poor. With the increase in the pulling speed (0.3 and 0.6 mm/s), as shown in Figure 9b,c, the surface flatness and uniformity of the sample are significantly improved, and there are no obvious appearance defects. However, as the pulling speed further increases (1 and 10 mm/s), as shown in Figure 9d,e, defects such as wrinkles and bulges appear on the surface, and the surface flatness and uniformity also significantly decrease.

**Figure 10:**
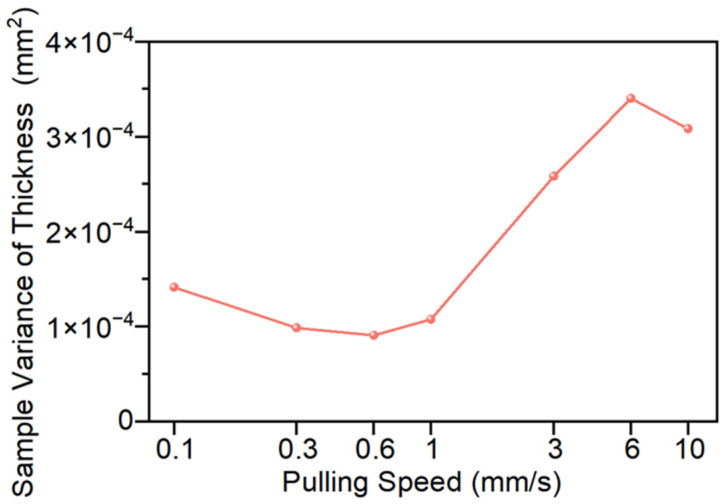
Thickness sample variances at different pulling speeds.

The influence of pulling speed on thickness uniformity is shown in Figure 10. Within the study range, the variance of thickness samples decreases first and then increases as the pulling speed increases. Among them, the variance of thickness samples is the smallest when the pulling speed is 0.6 mm/s, reaching 9.11 × 10^−5^ mm^2^, which is approximately 36% lower than that at 0.1 mm/s, and the thickness uniformity has significantly improved. However, when the pulling speed is 6 mm/s, the variance of thickness samples reaches the maximum, being 3.41 × 10^−4^ mm^2^, which is approximately 274% higher than that at 0.6 mm/s. However, when the pulling speed increases from 6 mm/s to 10 mm/s, the variance of thickness dimensions decreases, which may be due to the change in the material’s stress state under higher pulling speeds. At this time, the axial pulling force significantly increases, causing the fibers to be in a stretched state and basically remain straight, thereby improving the thickness uniformity.

**Figure 11:**
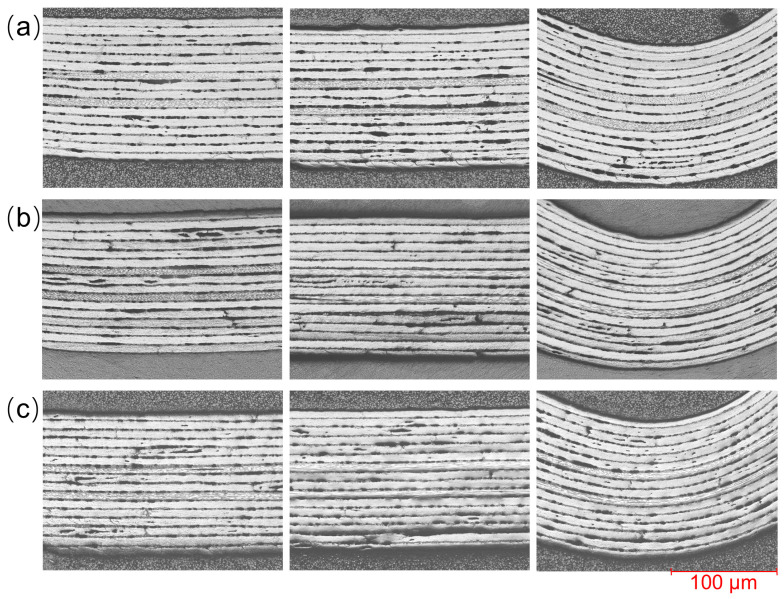
Metallographic microstructure photos at different pulling speeds: (**a**) 0.3 mm/s, (**b**) 0.6 mm/s, (**c**) 1 mm/s.

The influence of pulling speed on internal quality is shown in Figure 11. The experiment only analyzed the samples formed under the pulling speed conditions (0.3–1 mm/s) that had relatively better appearance quality and thickness uniformity. As shown in Figure 11a, when the pulling speed is 0.3 mm/s, the adhesion between the layers within the sample is poor, and certain fiber wave-like undulations and bending phenomena can be observed inside the sample. As shown in Figure 11b, when the pulling speed is 0.6 mm/s, the layer adhesion of the sample is relatively better, but it is not much different from the former case, and it is not ruled out that it is caused by sample preparation. As shown in Figure 11c, when the pulling speed is increased to 1 mm/s, the internal quality of the sample is also not much different from the previous two cases, and the wave-like undulation phenomenon of fibers in some local areas is more obvious.

The mechanism of the influence of the pulling speed on the mass of the prefabricated body is mainly reflected in the matching relationship between the deformation response time of the material and the allowable time of the process. When the pulling speed is relatively slow, the material is in a quasi-steady-state flow equilibrium state, which is more sensitive to sudden external disturbances (such as the start and stop of the pulling process and minor fluctuations in speed), and is prone to local deformation fluctuations. When the pulling speed is too fast, the local area of the material is difficult to adjust the deformation state in time, thereby increasing the possibility of defect occurrence. On the one hand, the resin does not have enough time to flow fully, thus resulting in local areas where resin is enriched or scarce. On the other hand, the interlayer slip and thickness redistribution process is difficult to fully complete, and the initial layup error and local strain concentration are retained and amplified.

In conclusion, within the scope of this study, the influence of the pulling speed on the preforming quality also shows a trend of “improvement first, then deterioration”. Among them, when the pulling speed is 0.3–0.6 mm/s, the appearance quality and internal structure of the sample are relatively stable with no significant difference, indicating that this range is a relatively reasonable pulling speed range. The thickness uniformity is the best at 0.6 mm/s, so it shows the relatively optimal preforming quality when comprehensively considering various quality indicators.

#### 4.2.3. Effect of Tension

**Figure 12:**
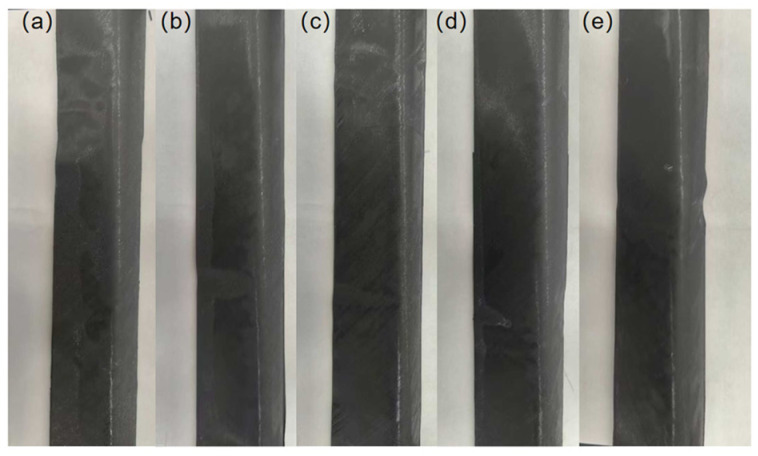
Photos of appearance quality at different tensions: (**a**) 10 N, (**b**) 20 N, (**c**) 30 N, (**d**) 40 N, (**e**) 50 N.

The influence of tension on the appearance quality is shown in Figure 12. Different tension levels were selected to study the sample areas for appearance comparison. Within the study range, the appearance quality generally shows a trend of “improvement first, then deterioration” as the tension increases. As shown in Figure 12a, the preforming sample with a tension of 10 N has more irregular waves and poor surface flatness and uniformity. When the tension is 20–40 N, as shown in Figure 12b–d, the waves of the preforming sample are significantly reduced, and the surface flatness and uniformity are relatively improved; the differences in appearance quality at different tension levels are not significant, and the overall appearance is relatively stable. At this time, the material has been appropriately tightened without causing excessive stretching deformation, resulting in relatively better surface quality of the formed part. In contrast, as shown in Figure 12e, the sample with a tension of 50 N has a relatively obvious wave-like undulation, and the surface flatness and uniformity are reduced.

**Figure 13:**
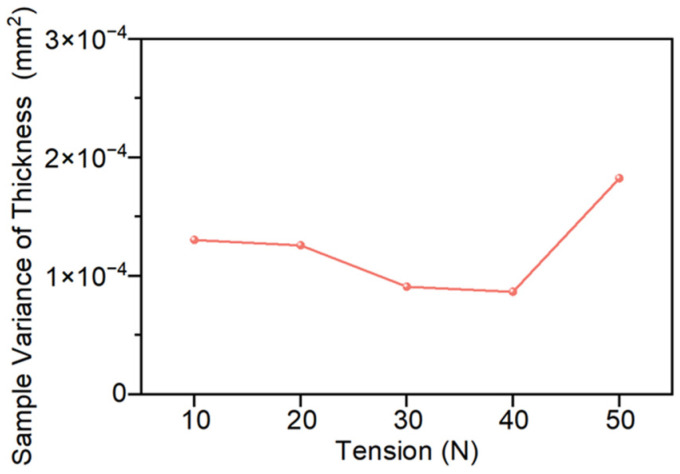
Thickness sample variances at different tensions.

The influence of tension on thickness uniformity is shown in Figure 13. Within the study range, the variance of thickness samples decreases first and then increases as the tension increases. When the tension increases from 10 N to 40 N, the variance of thickness samples decreases from 1.30 × 10^−4^ mm^2^ to 8.68 × 10^−5^ mm^2^, with a reduction of approximately 33%. This indicates that the thickness dispersion has significantly decreased and the overall uniformity has improved. However, when the tension continues to increase to 50 N, as shown in Figure 13, the overall variance increases to 1.82 × 10^−4^ mm^2^, which is approximately 110% higher than that at 40 N, and even greater than the overall variance of the molded part at 10 N.

**Figure 14:**
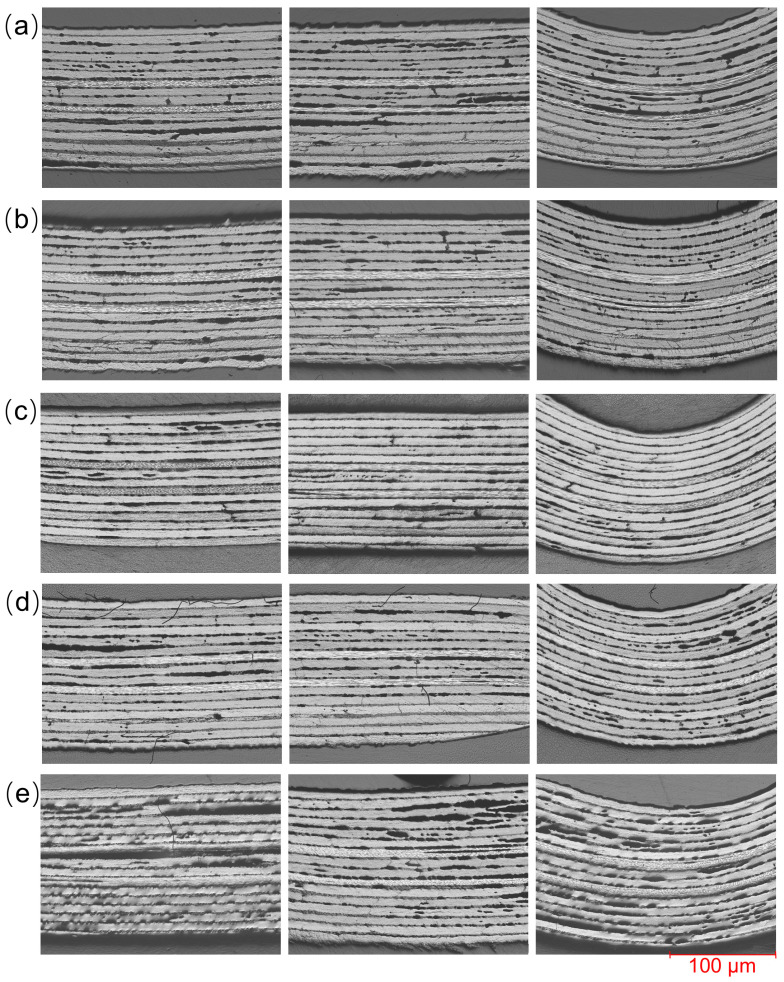
Metallographic microstructure photos at different tensions: (**a**) 10 N, (**b**) 20 N, (**c**) 30 N, (**d**) 40 N, (**e**) 50 N.

The influence of tension on internal quality is shown in Figure 14. Within the study range, the internal quality generally shows a trend of “improvement first, then deterioration” as the tension increases. As shown in Figure 14a, when the tension is 10 N, the overall structure of the laminate is relatively loose, and there is a certain degree of wavy fluctuation in the interlayer fibers. When the tension is 20–40 N, as shown in Figure 14b–d, the internal structure of the sample laminate improves significantly, and the wavy fluctuations of the fibers are significantly reduced. The internal quality under different tension conditions within this range is extremely similar and is generally in a stable state. When the tension is further increased, as shown in Figure 14e (tension of 50 N), the internal quality of the laminate significantly decreases, and the layer interface stratification and fluctuation are more obvious, and the interface continuity and uniformity are significantly reduced.

The above law can be attributed to the regulation of tension on the stress state and coordinated deformation ability of prepreg. When the tension is too small, the prepreg material is difficult to maintain a fully taut state and will show a sagging phenomenon before entering the preforming mold, thus making it easy to have initial defects during feeding. Moreover, when the tension is too low, the tension level of the material is too low, and the prepreg is not evenly stressed in each area as it passes through the mold, which is prone to local accumulation or insufficient flow, further exacerbating the formation of defects. On the other hand, when the tension is too high, the fibers are subjected to excessive stretching in the forming direction, restricting the necessary inter-laminar slip and structural adjustment of the laminate during deformation. This causes the prepreg material to be in an overly taut state as a whole, making it difficult to achieve coordinated deformation with the mold surface, thereby reducing the quality of preforming.

In conclusion, within the scope of this study, the influence of tension on the preforming quality also shows a trend of “improvement first, then deterioration”. Specifically, when the tension is within the range of 20–40 N, the appearance quality and internal structure of the sample are relatively stable and there are no significant differences, indicating that this range is a more reasonable tension range; meanwhile, at 40 N, the thickness uniformity is the best, so when considering all quality indicators comprehensively, it shows the relatively optimal preforming quality.

#### 4.2.4. Evaluation of Preforming Quality Under Optimal Process Parameters

In the above single-factor analysis process, the optimal parameter combination of preforming temperature, traction speed and tension has been obtained. Therefore, in this section, based on this optimal parameter condition, the forming quality of the preforming piece is comprehensively evaluated to verify the effectiveness and stability of the determined process parameters.

Based on the experimental results of overall appearance quality, thickness uniformity, and internal quality, it can be seen that under the optimal process parameters, the overall forming quality of the preformed part is good. Specifically, the surface of the preformed part is smooth and uniform, no obvious scratches, gelomas, or depressions are observed, meeting the required appearance requirements; the thickness distribution is uniform, and all measurement points meet the control requirements of ±5%; the internal structure is relatively dense and uniform, no obvious fiber folds or layering and other typical defects are observed, meeting the internal quality requirements. In conclusion, under the optimal process parameter combination, all evaluation indicators meet the expected requirements, indicating that this optimal process parameter combination can effectively improve the preformed quality and verifying the rationality of the aforementioned process parameter analysis.

### 4.3. Outlook and Future Work

On the basis of this work, future research can be further carried out from the following two aspects:

(1) Expansion of the thermal–mechanical coupling model:

This paper focuses on the preforming stage, based on the constant temperature and low curing degree characteristics of this stage, a pure mechanical model is established to optimize and verify the mold surface. On this basis, the thermo–mechanical coupling model can be introduced in the next step to further consider the influence of temperature field on the material deformation behavior in the preforming process, so as to improve the accuracy of the model.

(2) Simulation–Experimental verification under multiple process conditions:

In this paper, the comparison of mechanical response before and after profile optimization is completed only under fixed process conditions. Subsequent work can be based on the upgraded thermo–force coupling model to conduct parametric simulations within a wider range of process parameters (temperature, traction speed and tension), obtain strain field data under different conditions, and quantitatively compare them with the experimental results to systematically verify the model’s predictive ability and optimize the selection of process parameters.

(3) Expansion of the method to advanced material system:

The research methods used in this paper have potential applicability in principle to advanced material systems such as hybrid fiber composites and nanomodified prepregs. These materials are more sensitive to interlayer slip, thickness uniformity, and microscopic interface conditions. By optimizing the local strain distribution and preforming quality during the preforming stage, these new materials can provide a reference for their application in advanced pultrusion molding. Its potential applicability awaits verification through subsequent experiments and simulations.

## 5. Conclusions

This paper focuses on the complex material deformation and the frequent occurrence of defects in the preforming stage of advanced pultrusion forming. Taking the composite material L-shaped component as the research object, through numerical simulation and experimental research, the preforming mold surface is designed and the process parameters are studied, thereby achieving effective control of the preforming quality. The main conclusions are as follows:

(1) A mold surface optimization method aiming at uniformizing the three-dimensional strain field was proposed. By reconstructing the mold surface, the strains in the thickness direction and within the plane were successfully controlled within 5%. This method significantly improved the deformation coordination of the prepreg and effectively reduced the risk of defect formation, providing theoretical support for the mold design of complex cross-sectional components.
(2) The study reveals that the effects of preforming temperature, traction speed and tension on the quality of preformed parts (appearance quality, thickness uniformity and internal quality) show a trend of “improvement first, then deterioration”. Within the scope of this study, the optimal process parameters are temperature 90 °C, speed 0.6 mm/s, and tension 40 N. At this time, the sample has a smooth appearance, uniform thickness distribution, few internal quality defects, and the pre-formed quality meets the requirements.

## Figures and Tables

**Table 1:** Single-factor experimental design for processing parameters.

Run	Preforming Temperatures ( °C)	Pulling Speed (mm/s)	Tension (N)
1	60, 70, 80, 90, 100	0.3	30
2	Optimal Value	0.1, 0.3, 1, 3, 6, 10	30
3	Optimal Value	Optimal Value	10, 20, 30, 40, 50

## Data Availability

The data supporting the findings of this study are available within the article. Additional data that are not publicly available due to confidentiality agreements can be obtained from the corresponding author upon reasonable request.

## Supplementary Materials

The following supporting information can be downloaded at: https://www.mdpi.com/article/10.3390/polym18101244/s1. Figure S1. Boundary condition diagram: (a) Step 1, (b) Step 2. Among them, axes with primed numbers (1′, 2′, 3′) denote the local coordinate system; unprimed axes (1, 2, 3) denote the global system; Figure S2. Mesh distribution in the preforming process; Figure S3. (a) Dynamic DSC curve, (b) characteristic parameter fitting curve, and (c) complex viscosity-temperature curve. Figure S4. Schematic illustration of thickness measurement point locations. Figure S5. Schematic of the geometric model for prepreg deformation. Figure S6. (a) LE33 stress distribution cloud plot and (b) experimental thickness bar plot under the layup condition of [±45°] s. Figure S7. (a) LE33 stress distribution cloud plot and (b) experimental thickness bar plot under the layup condition of [0, 90] s. Figure S8. (a) LE33 stress distribution cloud plot and (b) experimental thickness bar plot under the layup condition of [0, 45] s. Figure S9. (a) LE33 stress distribution cloud plot and (b) experimental thickness bar plot under the layup condition of [0, −45] s. Table S1. Properties of EH919-34%-12KHF40C-U-194gsm. Table S2. Equivalent logarithmic strain under each experimental condition.

## Abbreviations

The following abbreviations are used in this manuscript:

ADP	Advanced pultrusion
PTFE	polytetrafluoroethylene
LE_11_	Logarithmic strains parallel to the fiber direction
LE_22_	Logarithmic strain perpendicular to the fiber direction
LE_33_	Logarithmic strain in the thickness direction
LE_12_	shear logarithmic strain
CSLIPEQ	equivalent slip measure
