# Supplementary material for: Mold Surface Optimization and Process Parameter Investigation for Preforming in Advanced Pultrusion of Composite Structures
Source: Polymers (Basel). 2026 May 20;18(10):1244. doi: 10.3390/polym18101244 (PMC13210426; doi:10.3390/polym18101244)
Supplement: Supplementary file 1 [file polymers-18-01244-s001.zip › polymers-4300433-supplementary.pdf]

Supplementary Materials:

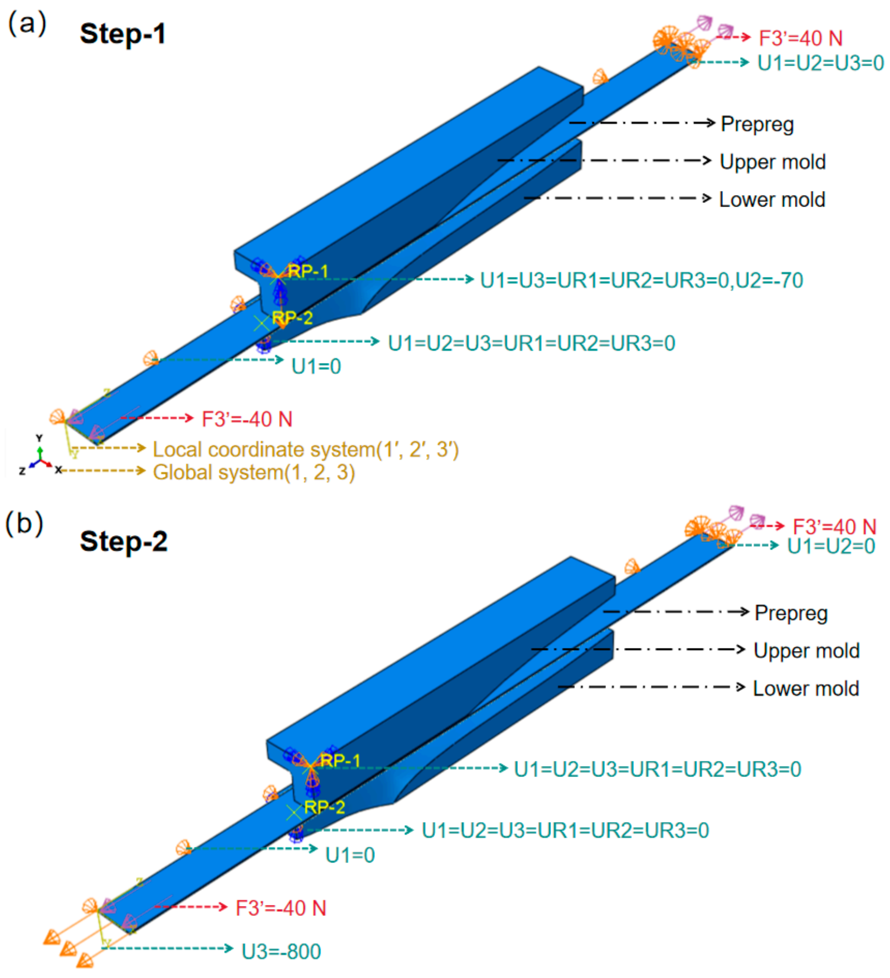

Figure S1. Boundary condition diagram：(a) Step 1, (b) Step 2. Among them, axes with primed numbers (1′, 2′, 3′) denote the local coordinate system; unprimed axes (1, 2, 3) denote the global system.

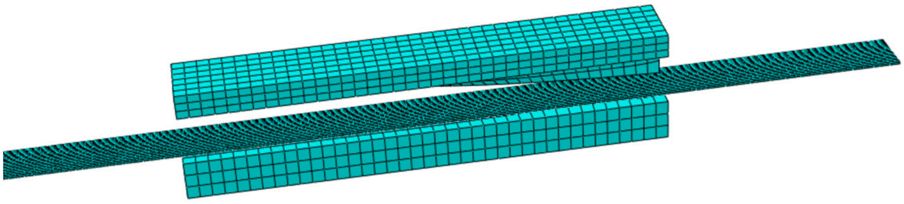

Figure S2. Mesh distribution in the preforming process.

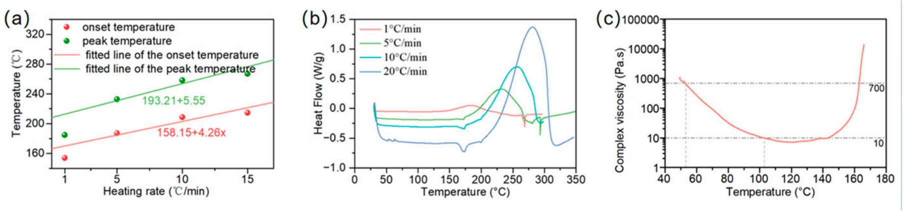

Figure S3. (a) Dynamic DSC curve, (b) characteristic parameter fitting curve, and (c) complex viscosity-temperature curve.

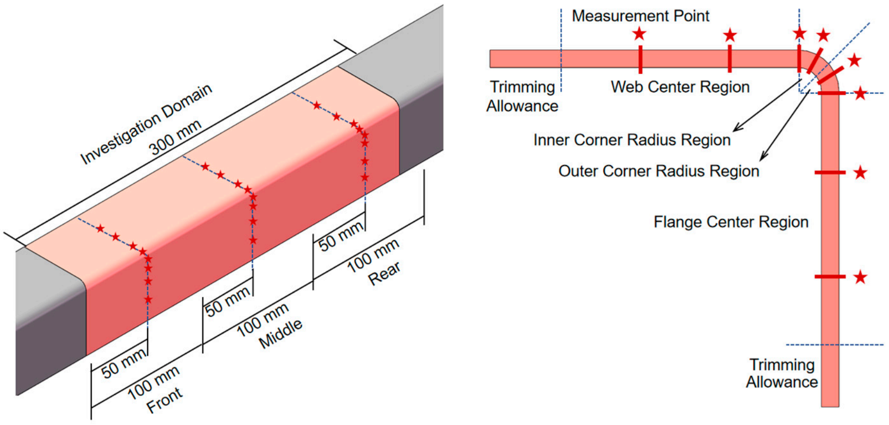

Figure S4. Schematic illustration of thickness measurement point locations.

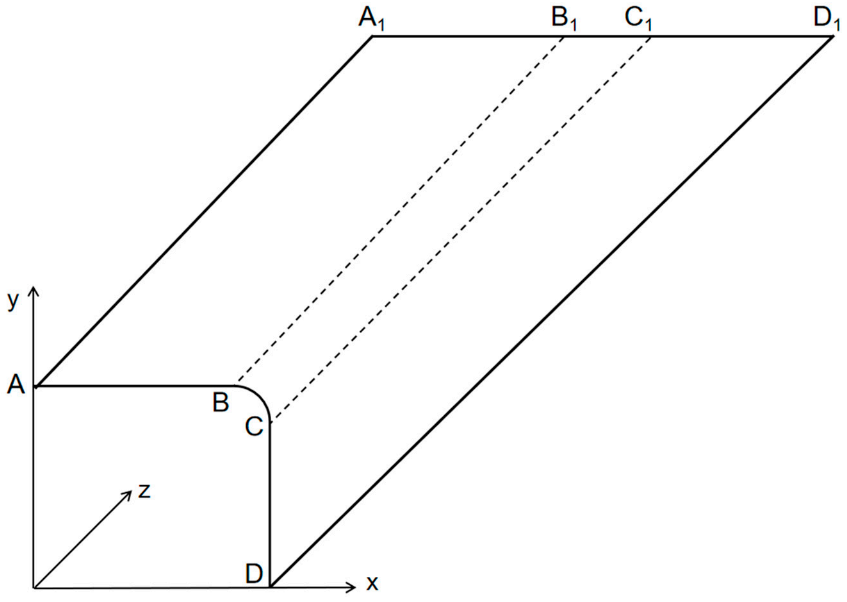

Figure S5. Schematic of the geometric model for prepreg deformation.

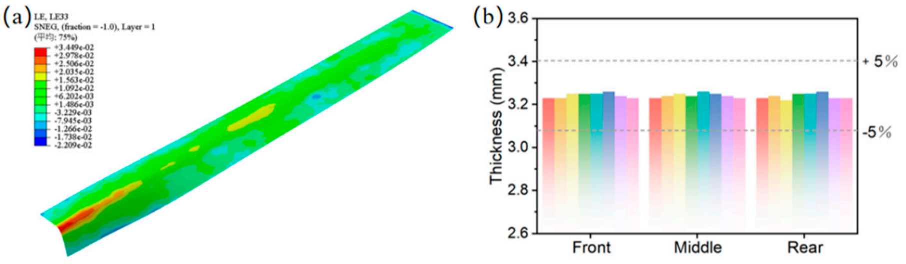

Figure S6. (a) LE_{33} stress distribution cloud plot and (b) experimental thickness bar plot under the layup condition of [±45°]s.

Under the [±45°]s layup, the maximum positive strain of LE_{33} was approximately 4.54%, and the minimum negative strain was approximately -1.31%, both within the ±5% range. According to the method described in Section 4.1.2, the measured thicknesses of the preformed parts were all distributed within the range of the reference thickness plus or minus 5%, and no abnormal points exceeding the

range were observed. Additionally, by comparing the simulation results with the experimental results, it can be found that the thickness distribution trend measured in the experiment is roughly consistent with the thickness strain distribution in the simulation, which to some extent verifies the reliability of the simulation model and the prediction results.

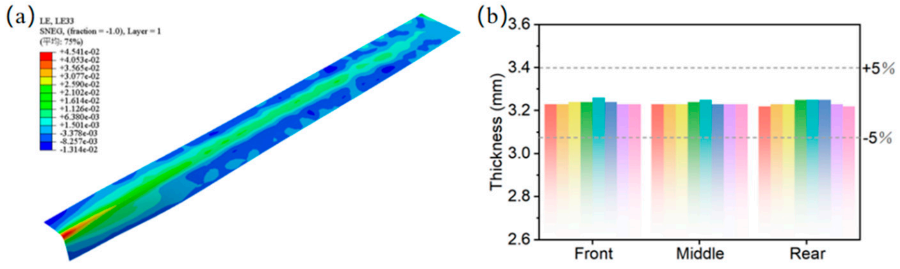

Figure S7. (a) LE_{33} stress distribution cloud plot and (b) experimental thickness bar plot under the layup condition of [0,90]s.

Under the layering of [0, 90]s, the maximum positive strain of LE_{33} was approximately 3.45%, and the minimum negative strain was approximately -2.21%, both within the range of ±5%. Additionally, the measured thicknesses of the preformed parts were all distributed within the range of the reference thickness ±5%, and no abnormal points exceeding the range were observed. These results are largely consistent with the simulation results, thereby confirming the reliability of the simulation model and the prediction results.

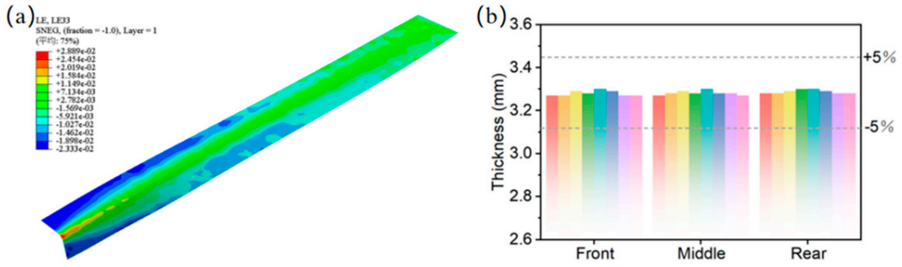

Figure S8. (a) LE_{33} stress distribution cloud plot and (b) experimental thickness bar plot under the layup condition of [0,45]s.

Under the [0, 45]s layup condition, the maximum positive strain of LE_{33} was approximately 2.89%, and the minimum negative strain was approximately -2.33%, both within the range of ±5%. The measured thicknesses were all distributed within the interval of the reference thickness ±5%, and no abnormal points exceeding the range were observed. This is consistent with the thickness strain distribution in the simulation, further confirming the reliability of the simulation model and the prediction results.

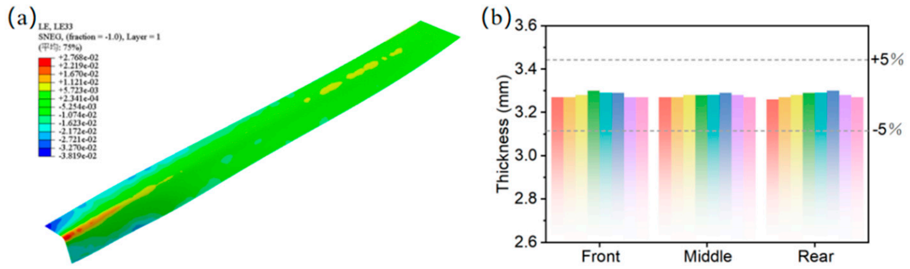

Figure S9. (a) LE_{33} stress distribution cloud plot and (b) experimental thickness bar plot under the layup condition of [0,-45]s.

Under the [0, -45]s layup condition, the maximum positive strain of LE_{33} was approximately 2.77%, and the minimum negative strain was approximately -3.82%, both within the ±5% range. The measured thicknesses were all within the interval of the reference thickness ±5%, and no abnormal points exceeding the range were observed. These results are basically consistent with the simulation results, thereby confirming the reliability of the simulation model and the prediction results.

Table S1. Properties of EH919-34%-12KHF40C-U-194gsm.

ρ	E1	E2	E3	V12	V13	V23	G12	G13	G23
**g/cm³**	**MPa**	**MPa**	**MPa**				**MPa**	**MPa**	**MPa**
1.56	160000	8300	8300	0.302	0.302	0.45	4910	4910	3437

Table S2. Equivalent logarithmic strain under each experimental condition.

**Process Conditions**			**Experimental results**	
**Preforming Temperature**	**Pulling Speed**	**Tension**	**$LE_{33\ min}$**	**$LE_{33\ max}$**
℃	mm/s	N	%	%
60			1.39	-0.72
70			0.87	-0.96
80	0.3	30	0.57	-0.65
90			0.53	-0.38
100			0.42	-0.79
	0.1		0.48	-0.74
	0.3		0.53	-0.38
	0.6		0.52	-0.40
90	1	30	0.52	-0.71
	3		0.89	-0.64
	6		1.71	-1.06
	10		1.26	-0.93
		10	0.91	-0.61
		20	0.92	-0.29
90	0.6	30	0.52	-0.40
		40	0.68	-0.55
		50	0.75	-0.78

Note: The reference thickness t_0 is the average of the thicknesses at multiple measurement points under each set of process conditions. $LE_{33\ min}=\ln(\frac{t\ min}{t\ 0})$, $LE_{33\ max}=\ln(\frac{t\ max}{t\ 0})$.
